# Case report: MRI and CT imaging features of a melanocytic tumour affecting a cervical vertebra in an adult dog, and review of differential diagnosis for T1W-hyperintense lesions

**DOI:** 10.3389/fvets.2024.1334813

**Published:** 2024-04-09

**Authors:** Elli Elizabeth Michaelidou, Adriana Kaczmarska, Rodrigo Gutierrez-Quintana, Joanna Morris, Gawain Hammond, Ana Cloquell

**Affiliations:** Institute of Biodiversity, Animal Health and Comparative Medicine, University of Glasgow, Glasgow, United Kingdom

**Keywords:** melanocytic neoplasia, Melan-A, T1W hyperintensity, cervical vertebra, melanoma of unknown primary, melanotic schwannoma, case report

## Abstract

A 7-year-old Lhasa Apso presented with a history of left thoracic limb lameness and neck pain. Magnetic resonance imaging revealed a well-defined, extradural lesion that was hyperintense on T1-weighted (T1W) images and isointense on T2-weighted (T2W) images and T2* images located at the left lamina of the C4 vertebra. Computed tomography showed an isoattenuating and contrast-enhancing mass centered on the left C4 vertebral lamina with associated osteolysis. The mass was surgically debulked, and histopathology revealed a malignant melanocytic tumour. The patient recovered completely and received radiotherapy and three doses of the melanoma vaccine as adjunctive treatment. Eighteen months following treatment, the patient presented with neck pain again, but further investigations were declined at this stage, and the patient was euthanised. To the author’s knowledge, this is the first case report describing the imaging characteristics of a cervical extradural melanocytic tumour in a dog. This case illustrates the MRI and CT imaging features and treatment of a canine melanocytic tumour of the cervical vertebrae.

## 1 Introduction

Primary melanocytic tumours of the central nervous system (CNS) are rare pathologies, with metastatic melanocytic tumours being more frequent (1). In humans, the majority of melanocytic tumours display characteristic signal patterns on MRI showing T1 and T2 shortening, resulting in T1W hyperintensity and T2W hypointensity (2). In general, apart from lipids, methaemoglobin and proteinaceous material, this T1W hyperintense appearance of the lesion is rarely observed on MRI (3) and only a few substances amongst which melanin, can create this T1W hyperintensity of the lesion (4). This case report describes the imaging findings and treatment of a melanocytic tumour affecting the cervical vertebra in an adult dog and a systematic approach to T1W hyperintense lesions on MRI.

## 2 Case description

**Figure 1:**
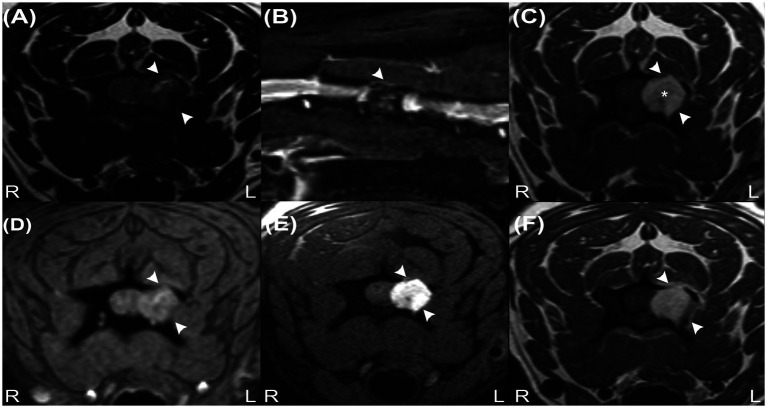
Transverse T2W image **(A)**, sagittal STIR image **(B)**, transverse precontrast T1W images **(C)**, transverse T2* image **(D)**, transverse T1W fat-suppressed precontrast image **(E)** and transverse contrast-enhanced T1W image **(F)** revealing an extradural mass at the level of the C4 vertebra (arrowheads). Note the characteristic hyperintense signal on image **(C)** (asterisk) and signal suppression on image **(B)** but absence of signal suppression on image **(E)**, indicating that the lesion does not contain fat tissue. The absence of signal voids on image **(D)** reveals that haemorrhage and calcifications are unlikely to be present in the mass.

A 7-year-old, 6.6 kg, male-neutered Lhasa Apso presented with a 3-month history of progressive left thoracic limb lameness and episodes of yelping. On presentation, the physical examination was unremarkable. Neurological examination revealed mild lameness and reduced postural reactions of the left thoracic limb, with severe pain on ventroflexion of the neck. The remainder of the neurological examination was normal. Neuroanatomical localisation was consistent with a left-sided lesion of the C6-T2 spinal cord segments. Haematology and serum biochemistry were normal. The patient was anaesthetised for cervical magnetic resonance imaging (MRI) using a 1.5-Tesla system (Magnetom Essenza, Siemens, Camberley, United Kingdom; MRI acquisition protocol is summarized in Supplementary Table S1) with a head–neck coil. A solitary, rounded, 10 mm diameter extradural, well-defined mass lesion was observed at the level of the left lamina and pedicle of the C4 vertebra, causing osteolysis (Figure 1). The mass was isointense on T2-weighted (T2W) images (with a central hyperintense cleft) (Figure 1A), markedly hyperintense on T1-weighted (T1W) images (Figure 1C), and with no signal voids on T2* (Figure 1D). Although the mass was suppressed on the short tau inversion recovery (STIR) sequence, there was no suppression on the specific fat-saturation (FAT-SAT) sequences, excluding a lipid-containing lesion (Figures 1B,E). Due to marked T1W hyperintensity, subtraction images were used for further evaluation of the contrast enhancement, which was homogenous and marked, associated with a linear and irregular enhancement of the adjacent periosteum and meninges. The lesion was causing spinal cord compression at the level of C4. The C4 left nerve root appeared enlarged and T1W hyperintense, with contrast enhancement of the adjacent cervical epaxial muscle.

**Figure 2:**
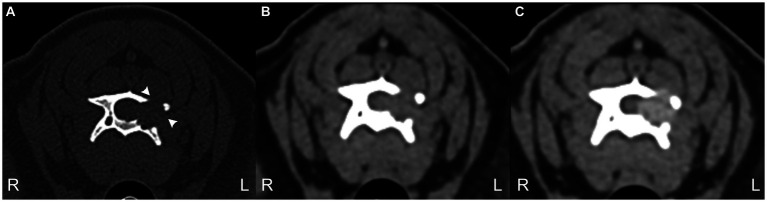
3 CT transverse images bone window **(A)** and soft tissue window precontrast **(B)** and post-contrast **(C)** at the level of C4 vertebra. Note the geographical bone resorption of the left lamina of C4 (arrowheads) and the moderate contrast enhancement of the mass. The mass demonstrated a mean of 58 Hounsfield units.

Given the suspicion of a melanin-containing lesion following the MRI, a careful examination of the skin, oral cavity, and eyes was performed, which did not reveal any lesions. Computed tomography (CT) of the entire vertebral column, thorax, and abdomen was performed using an 80-slice helical scanner with 120 kVp, 112 mAs, and 1.0 mm slice thickness (Aquilion Lightning, Canon, Duluth, United States). Images were reconstructed using medium- and high-frequency spatial algorithms after intravenous ioversol (Optiray 300 mg/mL, Guerbet, Roissy CdG, France) was administered (2 mL/kg) via a power injector (Injectron 82CT, MedTron AG, Germany) at a rate of 5 mL/s through the cephalic vein. CT demonstrated an osteodestructive, isoattenuating, well-demarcated, homogenously enhancing mass within the left lamina, also affecting the left pedicle and the cranial and caudal articular processes of C4 extending into the vertebral canal and into the left transverse foramen (Figure 2). Bone proliferation was not observed, nor was there evidence of metastatic disease or other primary neoplasms.

The patient underwent a left C4 hemilaminectomy, which revealed a black pigmented mass bulging through the C4 lamina. The left lamina of C4 and the articular process were removed, which allowed an en-block debulking of the mass. Although no infiltration or attachment to the meninges or nerve roots were observed, the ventrolateral aspect of the exposed vertebra following mass dissection retained a black discolouration, suggesting neoplastic infiltration. The cavitron ultrasonic surgical aspirator (CUSA: Integra LifeSciences Corporation, NJ, United States) was used at that level. As clear margins were not achievable, adjunctive radiotherapy was implemented 4 weeks after surgery (48Gy in 16 daily fractions). Histopathological and immunohistochemical examination of the mass revealed a well-differentiated infiltrating tumour with a high mitotic count (12 per 10HPF), positive for Melan-A, confirming a malignant melanocytic neoplasm. To delay metastatic spread, the patient also received three doses of the melanoma vaccine (Oncept, Merial Inc., Athens, GA, United States). The patient recovered after initial treatment, but 15 months following treatment, the patient exhibited an episode of neck pain. Further investigations at this stage were declined, and the patient was euthanised 18 months following the diagnosis. A post-mortem examination was declined.

## 3 Discussion

This case report presents a T1W hyperintense lesion of the cervical vertebra. On MRI, most substances and pathologies are hypointense on T1W images (3), as only a few naturally occurring substances are known to reduce T1 relaxation times (4), including; lipids, methaemoglobin, minerals (calcium, iron, copper, and manganese), proteins (including vasopressin-neurophysin II-copeptin complex), melanin, and gadolinium contrast agents (3, 4). Melanocytic tumours have been described as having the following imaging characteristics on MRI; T1W hyperintensity and T2W isointensity to hypointensity (5). In human literature, the paramagnetic properties of melanin are thought to be the reason behind these signal intensities (4). The melanocytic tumour in our case shares these typical MRI characteristics. Moreover, these have also been reported in a cat with an extradural melanoma within the lumbar region (6). However, there are reports indicating that this is not always the case. An example of this includes a recent case of an extradural melanoma affecting a thoracic vertebra in a dog, where the lesion was hyperintense on both T1W and T2W images (7). Similarly, a case of meningeal melanomatosis characterised by T1W hyperintensity and T2W hyperintensity with a few small areas demostrating T2W hypointensity has also been described in the literature (8). The cause of these atypical MRI findings is not completely understood. One of the current hypotheses includes alteration of the typical MRI signals by blood products from intratumoral haemorrhage (6).

**Figure 3:**
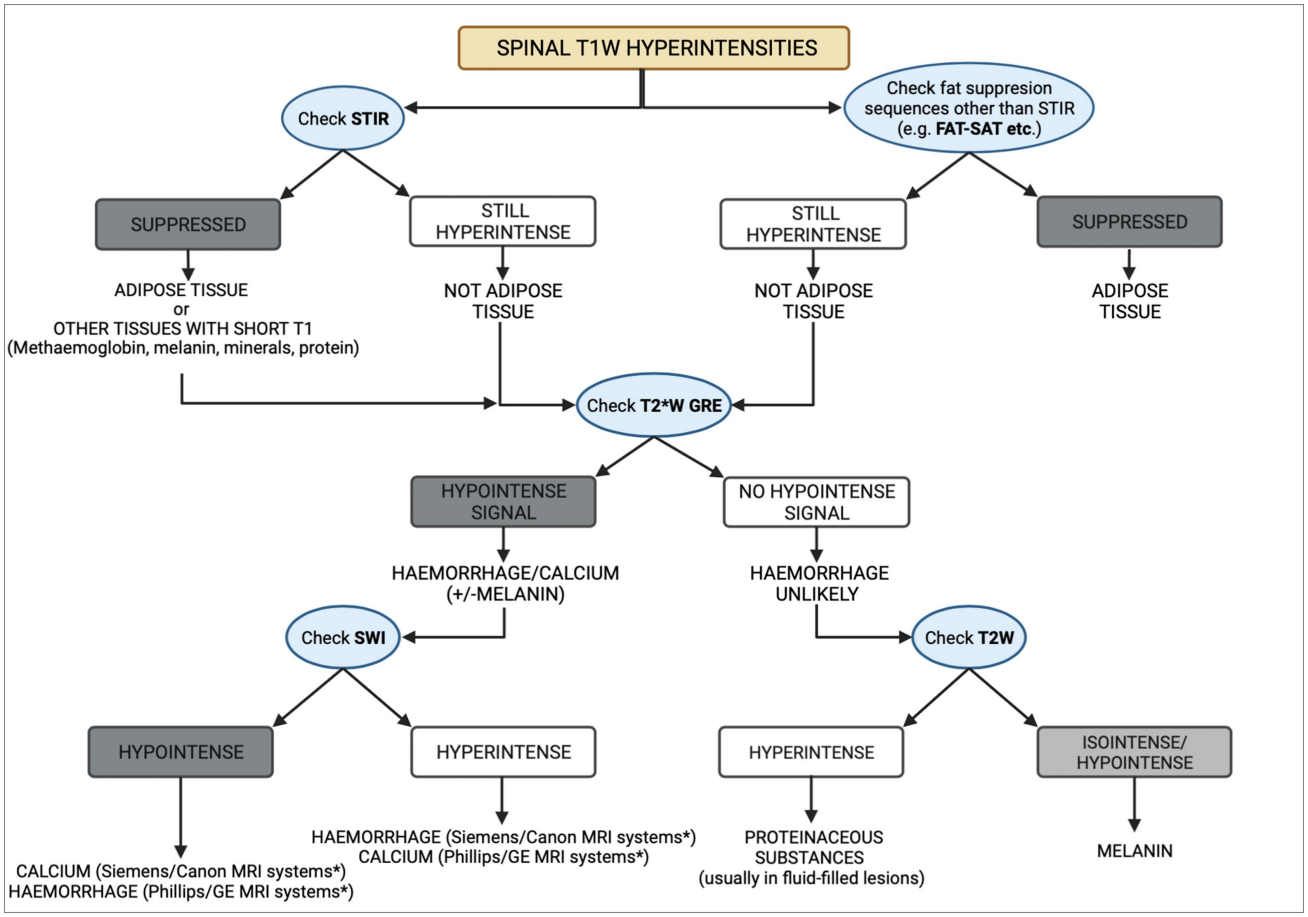
Diagram representing a methodical approach to the differential diagnoses of T1W spinal hyperintensities. CT can aid in the process, by taking into consideration the Hounsfield units of different substances. Asterisk (*) indicates the appearance of haemorrhage and calcium on susceptibility weighted imaging (SWI) studies which depends on the system manufacturer, with Siemens and Canon using a left-handed reference scheme and GE and Phillips using a right-handed reference scheme.

The differential diagnoses for a T1W hyperintense lesion can be narrowed using can be narrowed using localization and morphology of pathologies that cause the characteristic T1W hyperintensity, along with the evaluation of other MRI sequences or imaging techniques (3, 4). We propose a systematic approach detailed in Figure 3. The first step evaluates adipose tissue content using fat suppression sequences such as STIR and FAT-SAT (9). In this case, the mass’ signal was suppressed on STIR. However, as other tissues with short T1 relaxation may also be suppressed (10), fat-saturation was applied, showing a hyperintense mass, which excluded a lipid-containing lesion. To evaluate the content of haemoglobin and calcium, the T2* sequence was used. Signal voids on T2* were not observed, ruling out a calcified and likely haemorrhagic lesion. It is important to note that not all stages of haemorrhage will demonstrate a signal void on T2*. Oxyhaemoglobin, a diamagnetic substance (seen in the initial stages of haemorrhage), does not create signal voids, which in contrast to deoxyhaemoglobin and methaemoglobin, which have paramagnetic properties and induce faster T2* relaxation times, which result in signal voids (11–14). The CT characteristics supported these findings, as the Hounsfield units of the mass were not compatible with lipid, haemorrhage, or mineralization. Other minerals, such as iron, copper, and manganese, were considered unlikely, as their accumulation in the body is related to metabolic disturbances, usually creating bilateral and symmetrical parenchymal lesions (4). Similarly, based on the mass’ localisation, the presence of vasopressin was excluded (4, 15). Thus, a melanin-containing or proteinaceous lesion was the most likely cause of the T1W hyperintensity. Protein-containing lesions are usually hyperintense on T2W sequences. However, T2 relaxation times may vary depending on the amount of free water, protein content, and viscosity (3, 4). The T2W intensity in our case, suggested a melanin-containing lesion, which was considered more likely as it usually appears hypointense on these sequences (16). Despite the accuracy that may be achieved via evaluating all imaging sequences and diagnostic methods, histopathology remains the gold standard technique (17, 18). In this case report, the histopathological analysis and the positive result for Melan-A immunohistochemistry confirmed the presence of a melanocytic neoplasm.

Melanocytic neoplasms arise from melanocytes, which originate from the neural crest during embryogenesis and migrate to the skin, mucous membranes, and the CNS. Melanoma is common in dogs, usually involving the oral cavity, nailbed, eye, skin, and genitals (19, 20), with metastases reported in lymph nodes, the lung, brain, heart, spleen, and bone (20). Primary melanocytic tumours (melanocytoma, melanoma, melanocytosis, and melanomatosis) of the CNS are rare (1) but metastatic melanomas are more common (1, 21), with intracranial (22), intramedullary (23), and vertebral (24) involvement being documented in veterinary medicine. Additionally, primary CNS tumours such as schwannomas (25) and gliomas (26) may undergo melanisation. In human medicine, melanoma of unknown primary (MUP) refers to metastatic melanoma occurring in lymph nodes, subcutaneous tissue, or visceral sites in the absence of a detectable primary tumour (27). MUP may affect the vertebral column in humans (28–30), but it has not been documented in veterinary medicine.

Despite the imaging and histopathological findings in this case, it was not possible to differentiate between a MUP affecting the C4 vertebra (no primary lesion was found) or a melanotic schwannoma. Although MUP has not been previously described in dogs, there was no obvious involvement of the nerve roots or meninges observed during surgery, making melanocytic schwannoma less likely. Achieving a final diagnosis between melanotic schwannomas and melanocytic lesions can be challenging because both are histologically similar. Immunohistochemical stains are not always useful because all lesions generally express S-100 and one or more melanocytic markers. Stains for components of the basement membrane can be used to discriminate schwannomas, but overlapping staining patterns have been observed with other melanocytic lesions. Therefore, in human medicine, mutational analyses are being developed (31).

To the author’s knowledge, MRI and CT findings of a solitary melanocytic mass affecting the cervical vertebral column, without an identified primary site, have not been previously reported in dogs. These findings may provide the imaging characteristics for future recognition of melanin-containing masses, as well as a systematic approach to T1W hyperintense lesions. Although MRI can assist with the differential diagnosis, histopathological examination remains essential for a definitive diagnosis (32).

## 4 Conclusion

To the author’s knowledge, this case describes the first documented melanocytic neoplasia affecting the cervical vertebra in an adult canine and its surgical and medical management. This report provides the basis for recognizing such neoplasms in the future, as well as a diagram that can aid the clinician in narrowing down their differential diagnoses of a T1W hyperintense lesion in the spine.

## Data availability statement

The original contributions presented in the study are included in the article/Supplementary material, further inquiries can be directed to the corresponding author.

## Ethics statement

Ethical approval was not required for the studies involving animals in accordance with the local legislation and institutional requirements because this was a retrospective case report of a canine patient seen at the Neurology Department of Glasgow’s University Small Animal Hospital. The patient was referred for further veterinary care due to cervical pain and thoracic lameness. Written informed consent was obtained from the owners for the participation of their animals in this study.

## Author contributions

EM: Conceptualization, Software, Writing – original draft, Writing – review & editing. AK: Conceptualization, Supervision, Validation, Visualization, Writing – review & editing, Writing – original draft. RG-Q: Supervision, Validation, Visualization, Writing – review & editing, Writing – original draft. JM: Supervision, Validation, Visualization, Writing – review & editing, Writing – original draft. GH: Software, Supervision, Validation, Visualization, Writing – review & editing, Writing – original draft. AC: Conceptualization, Supervision, Validation, Visualization, Writing – review & editing, Writing – original draft.
