# Supplementary figures and images for: Case report: MRI and CT imaging features of a melanocytic tumour affecting a cervical vertebra in an adult dog, and review of differential diagnosis for T1W-hyperintense lesions
Source: Front Vet Sci. 2024 Apr 9;11:1334813. doi: 10.3389/fvets.2024.1334813 (PMC11035764; doi:10.3389/fvets.2024.1334813)

Supplementary Material


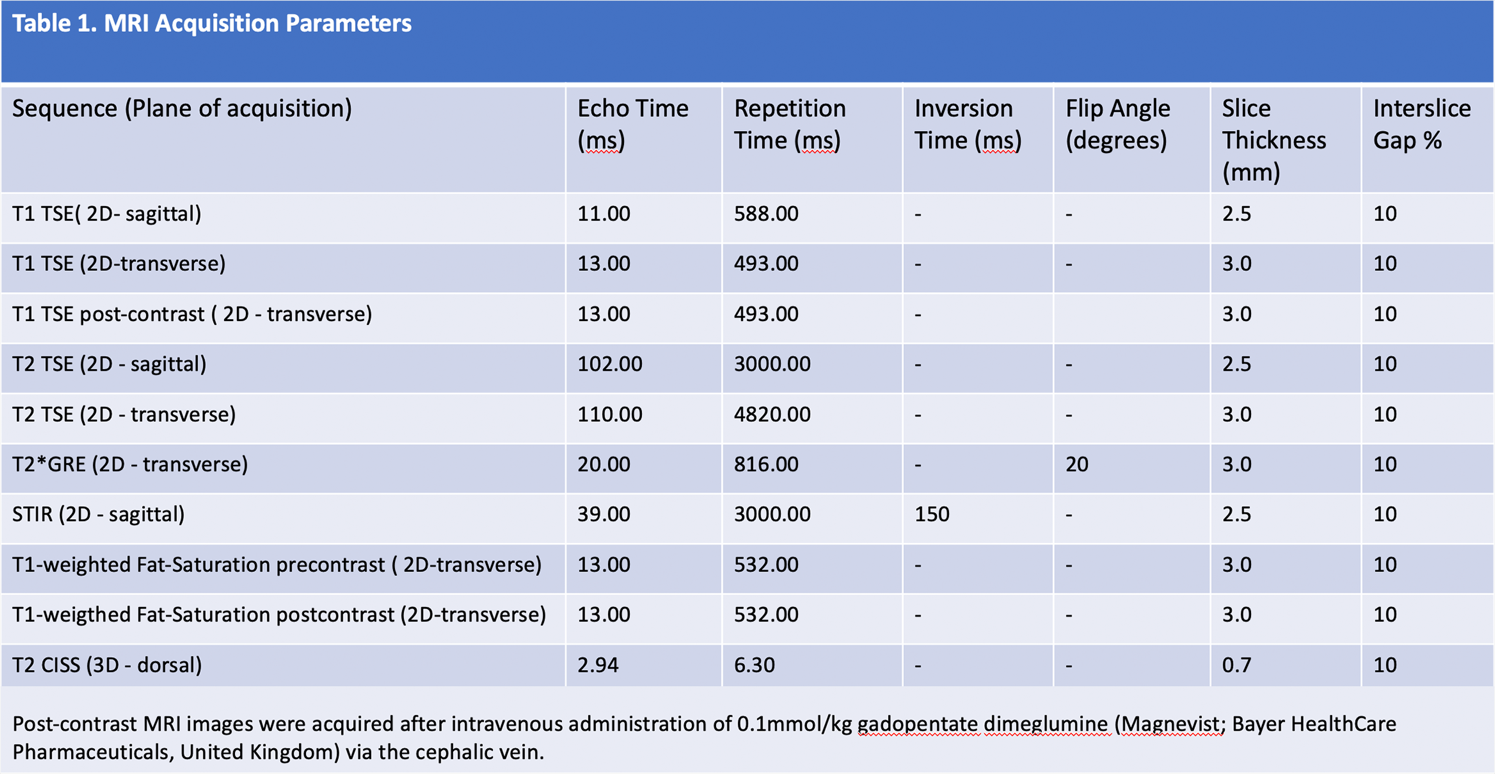

Supplement: Supplementary file 1 [file Table_1.docx]
